# Spatial distribution and influencing factors of high-quality tourist attractions in Shandong Province, China

**DOI:** 10.1371/journal.pone.0288472

**Published:** 2023-07-14

**Authors:** Min Wang, Shumin Liu, Chenxu Wang

**Affiliations:** 1 School of Geography and Tourism, Qilu Normal University, Jinan, China; 2 School of Geography and Tourism, Qufu Normal University, Rizhao, China; 3 School of Resources and Technology, Beijing Normal University, Beijing, China; Northeastern University (Shenyang China), CHINA

## Abstract

Optimizing the spatial layout of high-quality tourist attractions is of great significance in the sustainable development of the tourism industry. This work employs the ArcGIS spatial analysis tool to study the form, equality, and density of the spatial distribution of the 892 3A+ tourist attractions (high-quality tourist attractions hereafter) in Shandong Province, China. It also examines the factors influencing the spatial distribution of tourist attractions from the perspectives of geographic features and landscapes, culture and heritage, socioeconomic development, and transportation. We therefore find the following: 1) High-quality tourist attractions in Shandong Province have obvious clustering in spatial distribution with the high-density areas mainly concentrated in Qingdao, Jining, Jinan, Tai’an and other cities. Influenced by resource endowment and economic development, the two major geographical areas in Central Shandong and Jiaodong Peninsula have the most concentrated distribution of high-quality tourist attractions. 2) The distribution of high-quality tourist attractions shows a southwest‒northeast clustering direction; Qingdao is a high-high clustering area, and Heze is a low-high clustering area with low uniformity of spatial distribution and obvious spatial divergence. 3) Tourist attractions show an obvious "N" type high-density distribution belt and nuclear density distribution across the three existing agglomeration centers in the Jining–Tai’an intersection, Binzhou–Dongying intersection, and Qingdao Jiaozhou Bay coast. 4) Topography, climate conditions, history and culture are intrinsic factors affecting the spatial distribution of tourist attractions, while socioeconomic and transportation conditions are external requirements for the development thereof; collectively, they constrain the spatial distribution of high-quality tourist attractions.

## 1. Introduction

Tourist attractions are the foundation of regional tourism economic development. They are at the core of the tourism supply chain, as they provide tourism products and their spatial distribution affects strategic decision-making regarding regional tourism [1]. To promote the tourism industry, the former China National Tourism Administration issued the *Rating Standards for A-level Tourist Attractions*. Since then, China’s regional tourism authorities have invested significant efforts in promoting the ratings of local tourist attractions, which have been widely recognized by tourists [2, 3]. Tourist attractions with high ratings have thus become selling points; these attractions draw both domestic and international tourists [4].

Recently, the number of A-class tourist attractions in China has grown rapidly, the quality of these attractions has been optimized, and the number of tourists visiting these attractions has increased in an orderly manner [5, 6]. The distribution of tourist attractions and their influencing factors have been the typical focus of tourism geography and tourism planning [7, 8]. Other countries lack a rating system for tourist attractions similar to China’s. Tourism research abroad mainly focuses on resource assessment [9], marketing [10], market planning [11], demand forecasting [12, 13], tourists’ behaviors [14], and the management and marketing of tourist destinations [15]. Hence, there has been less research on tourism resources or the spatial evolution of tourist attractions. Tourism research in China is characterized by the following major features: (1) Theoretical research is being constantly enriched, and research methods are becoming increasingly mature [16]. Based on theoretical approaches such as the life cycle theory of tourism places [17], growth pole theory [18], actor network theory [19], and economic growth alliance theory [20], domestic scholars deeply analyze the collaborative relationships among multiple actors involved in tourism activities. Research methods are mainly quantitative, primarily mathematical models and GIS spatial analysis techniques [21, 22], such as the nearest neighbor index [23], Gini coefficient [24], kernel density analysis [25], and center of gravity shift [26]. Tools and techniques such as grid dimensional analysis, spatial autocorrelation, and geographic probes have also received wider application in recent years. (2) Tourism promotions focus on not only the tangible physical entities but also the perceived images of tourist attractions [27]. The shaping of tourism destination image and construction of tourism brand have become research hotspots in tourism geography, whose research methods mainly entail qualitative analysis and research content is primarily tourism destination marketing, tourism image competitiveness, and improvement paths, with micro case studies as their main focus [28]. (3) Tourists are the main body of consumption at tourist attractions. Tourist satisfaction comprises the perception and experience of tourists regarding tourism products and services and has become an important standard for measuring the service level of tourist attractions [29]. The existing research on tourist satisfaction started relatively late but has developed rapidly, showing a diversified trend in research subjects and a rather comprehensive evaluation system, thereby providing useful guidance for the construction of tourist attractions [30]. (4) Transportation is the channel connecting tourists and tourist attractions [31]. The accessibility of a tourist attraction is an important indicator for measuring the level of regional tourism. Scholars have attached importance to the relationship between the accessibility of tourism transportation and the spatiotemporal evolution pattern of tourist attractions [32]. Recently, China’s high-speed railway network has also undergone a large scale and high degree of modernization [33]. Since transportation modes have a certain impact on tourist attractions, the impact of this high-speed railway extension on regional tourism has become another focus of scholars. Overall, then, scholars focus on the factors affecting the natural environment, human history and economic industrial aspects of tourist attractions to analyze it’s speed of expansion and economic benefits.

Nevertheless, the extant domestic research on tourist attractions requires further elaboration. There are relatively numerous case studies of specific tourist attractions, but there is a lack of comprehensive regional research [34, 35]. The scope and scale of these research objects are also relatively limited, especially for large-scale and high-level regions, while there are few spatial analysis-oriented studies on tourist attractions. Tourism attractions’ ratings are based on their traffic, accessibility, safety and sanitation conditions, comprehensive management, etc. Of all the rated tourist attractions, its 3A+ tourist attractions (high-quality tourist attractions hereafter) comprehensively represent a region’s tourism development. These can more widely account for the salient rural and urban research areas and better reveal the spatial distribution characteristics and rules of tourist attractions, acting as a guide for regional efforts to expand a tourism market and for tourists to make travel decisions.

Shandong Province has particularly unique advantages in terms of natural and cultural tourism resources. By the end of 2020, it had 892 high-quality tourist attractions, ranking first among provincial-level units in China. From the formation of its "one mountain, one water and one saint" tourism route in the 1990s to its construction of the "Hospitality Shandong" cultural tourism brand at the beginning of this century, Shandong Province has ensured the basic conditions for the development of tourism. Recently, with the promotion of Confucian culture as their core, it has launched ten major cultural tourism destinations, and its influence in the country has been further enhanced. However, the ongoing development of tourism in Shandong Province also entails some challenges. For instance, the industry’s upgrades are slow, its structure must be optimized, its fundamentals are still weak, and its tourism products are still not attractive enough [36]. These issues can all be addressed through the spatial optimization of tourist attractions.

Accordingly, this paper employs ArcGIS 10.8 spatial analysis to examine the spatial structure of Shandong’s high-quality tourist attractions and to analyze the factors that shape this distribution. This study is therefore expected to provide meaningful theoretical support for the exploitation of Shandong’s tourism resources and the healthy growth of its provincial tourism industry.

## 2. Data source and research methods

### 2.1 Data source

**Fig 1:**
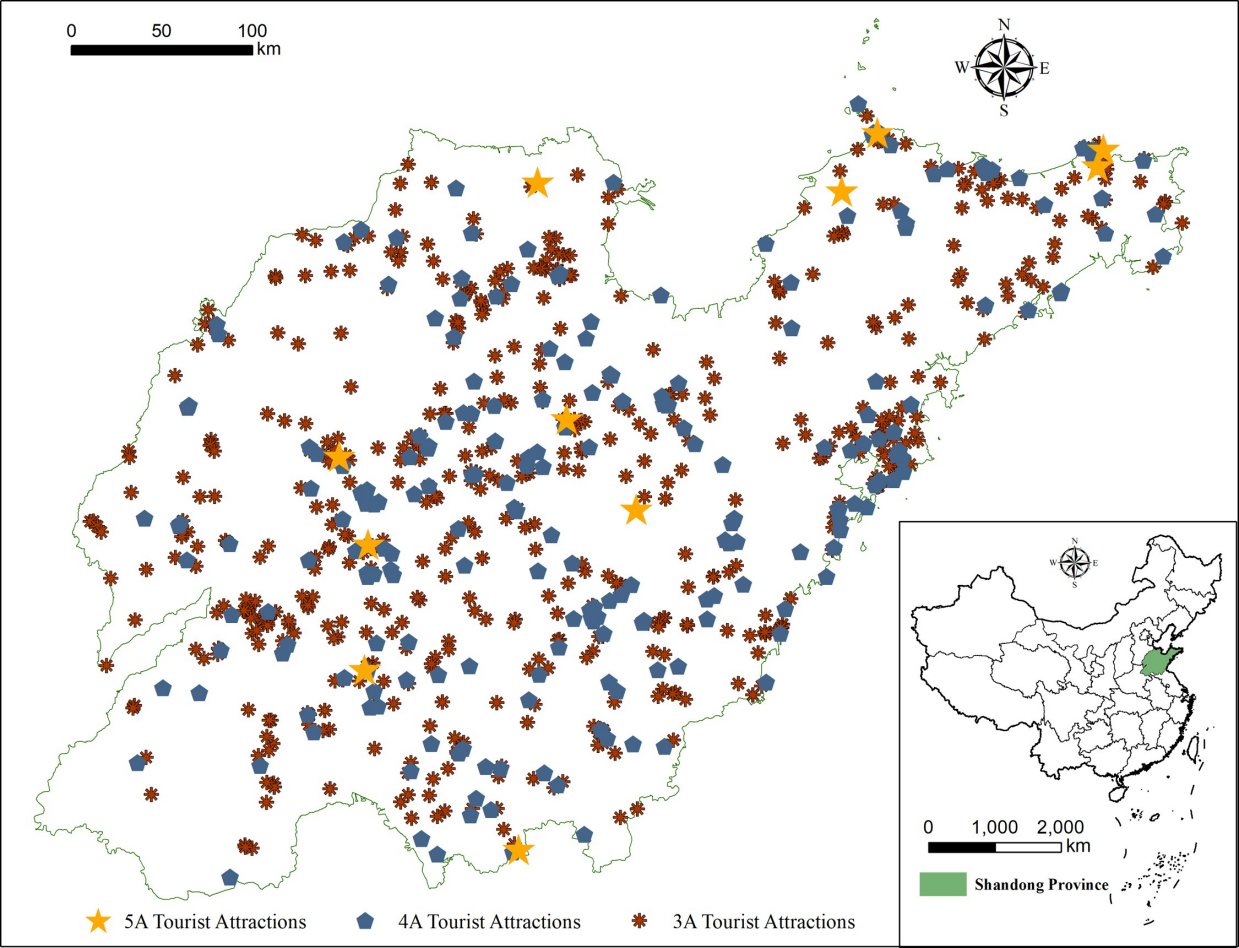
Spatial distribution of high-quality tourist attractions in Shandong Province.

The tourist attractions used in this research are detailed in the *List of A-class Tourist Attractions in Shandong Province*, available on the website of the Shandong Provincial Department of Culture and Tourism (whhly.shandong.gov.cn). This list was verified by reviewing the websites of the tourism departments of the 16 prefecture-level cities in Shandong. If a disparity was identified, further clarification was ensured through telephone interviews with these government agencies. The compiled data were then used in the analysis. The coordinates of each attraction are based on the location of its ticket office, which was obtained through webpages (jingweidu.bmcx.com) that provide these coordinates. In addition, there are socioeconomic and physical geographic data such as administrative region data, elevation data, river data, and road data in Shandong Province. The basic map data and the natural geography data are acquired from the Resources and Environment Science and Data Center of the Chinese Academy of Sciences (www.resdc.cn) [37]. The socioeconomic data are drawn from the *Shandong Statistical Yearbook 2021*. As of December 2020, there were thus 11 5A tourist attractions, 224 4A tourist attractions, and 657 3A tourist attractions in Shandong. The number of high-quality tourist attractions in each prefecture-level city is given in Table 1, and the distribution of these tourist attractions is shown in Fig 1.

**Table 1:** Number of high-quality tourist attractions in prefecture-level cities in Shandong Province.

City	Number of 5A	Number of 4A	Number of 3A	Total
	Tourist Attractions	Tourist Attractions	Tourist Attractions	
Qingdao	1	27	74	101
Linyi	1	26	70	97
Jining	1	24	71	95
Yantai	2	20	53	75
Weifang	1	16	46	63
Jinan	0	15	48	63
Binzhou	1	16	42	59
Tai’an	2	13	42	57
Zibo	1	12	38	51
Rizhao	1	13	32	46
Weihai	0	9	30	39
Dongying	0	9	29	38
Dezhou	0	8	26	34
Zaozhuang	0	5	27	32
Liaocheng	0	7	20	27
Heze	0	4	9	13

### 2.2 Main research techniques

#### 2.2.1 Nearest neighbor index

The nearest neighbor index employs complete spatial randomness (CSR) as the comparison criterion to derive the characteristics of the spatial distribution of points. Namely, the observed nearest distance is compared with the expected nearest distance. When the observed nearest distance is smaller than the expected nearest distance (assuming a random point distribution), the points tend to be spatially clustered; if the observed nearest distance is greater than the expected nearest distance, then the distribution is dispersed [25]. The formula to calculate the NNI is:

$$\text{R}\;=\;\frac{{\text{r}}_{\text{obs}}}{{\text{r}}_{\exp}}\;=\;\frac{\frac{\sum_{\text{i}=1}^{\text{n}}\;{\text{d}}_{\min\left({\text{S}}_{\text{i}}\right)}}{\text{n}}}{\frac{1}{\sqrt[{2}]{\frac{\text{n}}{\text{A}}}}}\;=\;\frac{\sqrt[{2}]{\rho }}{\text{n}}\;\sum_{\text{i}=1}^{\text{n}}{\text{d}}_{\min\left({\text{S}}_{\text{i}}\right)}$$ (1)

where R is the NNI value; r_obs_ is the observed average nearest distance; r_exp_ is the expected average nearest distance; A is the size of the study area; S_i_ represents each point; d_min_ is the distance between each point and its closest neighboring point; and n is the number of points in the study area.

1. If R = 1, namely, r_obs_ = r_exp_, the spatial distribution of points is random;
2. If R<1, namely, r_obs_ < r_exp_, the points tend to be spatially clustered;
3. If R>1, namely, r_obs_ > r_exp_, the points are dispersed. r_obs.

#### 2.2.2 Standard deviation ellipse

The standard deviation ellipse takes the four elements center, azimuth, major axis, and minor axis as parameters and quantitatively describes the characteristics of the spatial distribution of geographic elements. The long axis reflects the main spatial distribution trend of geographic elements, the short axis reflects the secondary distribution trend, the length of the long and short axes also reflects the degree of dispersion in the primary and secondary directions, and the ratio of the two reflects the discrete distribution pattern [23]. The formula is as follows:

$$\text{SD}E_{\text{x}}\;=\;\sqrt{\frac{\sum_{\text{i}=1}^{\text{n}}\;{\left({\text{x}}_{\text{i}}\;-\;\bar{\text{X}}\right)}^{2}}{\text{n}}}$$ (2)

$$SDE_{\text{y}}\;=\;\sqrt{\frac{\sum_{\text{i}=1}^{\text{n}}\;{\left({\text{y}}_{\text{i}}\;-\;\bar{\text{Y}}\right)}^{2}}{\text{n}}}$$ (3)

where x_i_ and y_i_ represent the coordinates of element i; $\left\{\bar{\text{X}},\bar{\text{Y}}\right\}$ represents the average center of the element; and n represents the total number of elements.

#### 2.2.3 Kernel density estimation

Kernel density estimation can estimate the distribution density of spatial elements and clearly indicate the dispersion or concentration thereof [24]. The formula is:

$${\hat{\lambda }}_{\text{h}}(\text{s})\;=\;\sum_{\text{i}=1}^{\text{n}}\;\frac{3}{\pi {\text{h}}^{4}}\;{\left[1\;-\;\frac{{\left(\text{s}-{\text{s}}_{\text{i}}\right)}^{2}}{{\text{h}}^{2}}\lambda \right]}^{2}$$ (4)

where s is the spatial position of an element; s_i_ represents the elements that fall within the space formed by using s as the center of the circle; and h is the position of the *i*^th^ spatial element.

#### 2.2.4 Spatial autocorrelation analysis

Using the global spatial autocorrelation Moran’s I index to measure the spatial distribution of high-quality tourist attractions in Shandong Province, we analyze the distribution of high-quality tourist attractions in Shandong Province from the overall macro perspective to verify the correlation between high-quality tourist attractions and surrounding spatial units [26]. The formula for the correlation coefficient is:

$$\text{I}=\frac{\text{n}\;\sum_{\text{i}=1}^{\text{n}}\;\sum_{j=1}^{\text{n}}\;{\text{w}}_{\text{ij}}{\text{z}}_{\text{i}}{\text{z}}_{\text{j}}}{\left(\sum_{\text{i}=1}^{\text{n}}\;\sum_{j=1}^{\text{n}}{\text{w}}_{\text{ij}}\right)\;\sum_{\text{i}=1}^{\text{n}}\;{\text{z}}_{\text{i}}^{2}}$$ (5)

Where w_ij_ represents the spatial weight between elements i and j, z_i_ represents the deviation of the attribute of element i from its average value $\left({\text{x}}_{i}-\bar{\text{x}}\right)$, and n is the number of all elements. Moran’s I index ranges from -1 to 1. The larger the absolute value is, the stronger the spatial correlation.

1. Moran’s I >0, research objects are positively correlated in space;
2. Moran’s I >0, research objects are not spatially correlated and tend to be randomly distributed;
3. Moran’s I >0, research objects are negatively correlated in space.

#### 2.2.5 Climate comfort assessment model

Climatic conditions are an important factor influencing the development of regional tourism. W.H. Terjung was the first to propose the human climate comfort index, which has been continuously revised by scholars and remains widely used in related research [38]. In this study, we obtained data on temperature, humidity, sunshine hours and wind speed from the National Meteorological Science Data Center (data.cma.cn) to evaluate the climate comfort of different cities in Shandong Province [39]. The temperature and humidity index mainly reflects the human body’s perception of temperature and humidity (Eq 6), while the wind efficiency index accounts for the influence of temperature, wind speed and sunlight on the human body’s temperature perception (Eq 7); these indices are calculated as follows [40]:

THI=(1.8×T+32)−0.55×(1−f)×(1.8×T−26) (6)

K=−(10.9V+10.45−V)(33−T)+8.55S (7)

where THI is the temperature and humidity index, K is the wind efficiency index, T is the temperature (°C), f is the relative humidity (%), V is the wind speed, and S is the sunshine hours (h).

Since Shandong Province has four distinct seasons, both land and sea, and complex topographic conditions, the temperature and humidity index and wind efficiency index are combined to more specifically assess the climate comfort of different cities [41, 42]. The temperature and humidity index is used in the summer half-year, and the wind efficiency index is used in the winter half-year, as shown in Table 2.

**Table 2:** Classification of climate comfort index levels.

Feel extent	Temperature and humidity index (Summer)	Wind efficiency index (Winter)
Extremely cold	<14.0	<-800
Cold	14.0∼18.0	-800∼-600
Comfortable	18.0∼24.5	-600∼-200
Hot	24.5∼25.0	-200∼-10
Sultry	>25.0	>-10

## 3. Results

### 3.1 Characteristics of the high-quality tourist attraction distribution

#### 3.1.1 Spatial distribution form

The spatial distribution of point elements has three forms: random, regular, and clustered. The occurrences of these forms often alternate, increasing the difficulty of distinguishing them. The observed nearest distance is r_obs_ = 45.41 km, while the expected nearest distance is r_exp_ = 84.12 km, and the NNI R = 0.54<1 (Table 3). Therefore, the distribution of high-quality tourist attractions in Shandong exhibits a clustering pattern.

**Table 3:** Results of nearest distance analysis.

Number of High-Quality Tourist Attractions	Observed Nearest Distance (km)	Expected Nearest Distance (km)	NNI (R)	Form of Spatial Distribution
892	45.41	84.12	0.54	Clustered

According to the differences in geographical location, topography, historical culture, and economic development among the different cities in Shandong Province, this study divides Shandong Province into five geographical regions: Northwest Shandong (Dezhou, Binzhou, Liaocheng, Dongying), Southwest Shandong (Jining, Heze), Central Shandong (Jinan, Taian, Zibo, Weifang), Southern Shandong (Linyi, Zaozhuang, Rizhao), and Jiaodong Peninsula (Qingdao, Yantai, Weihai). Therefore, during calculations with Excel software, the N value is taken as 5, the Gini coefficient of tourist attraction distribution 0.90, and the uniformity of distribution C = 0.10, indicating that high-quality tourist attractions in Shandong Province are concentrated and distributed with low uniformity.

#### 3.1.2 Spatial autocorrelation analysis

**Fig 2:**
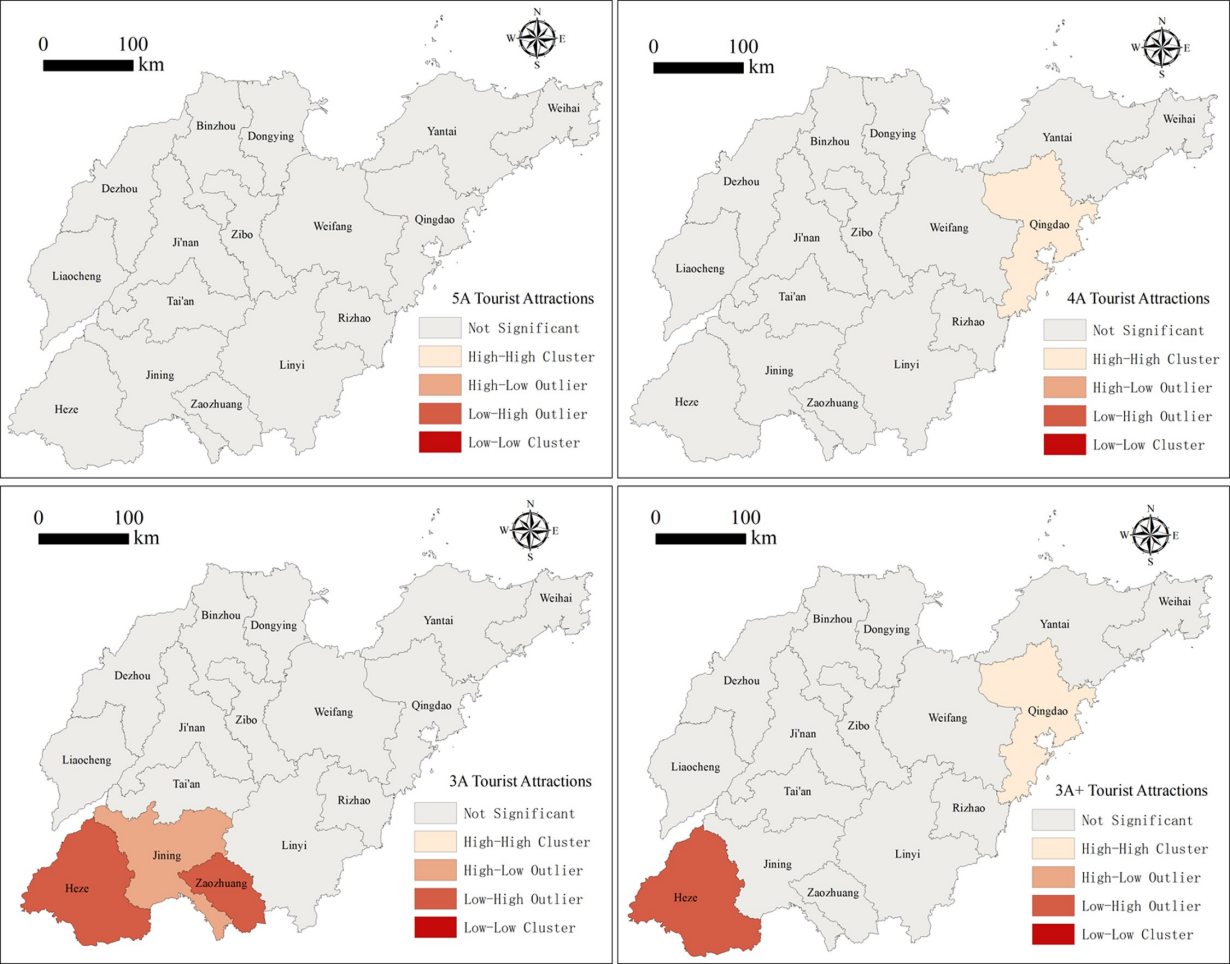
Spatial distribution of the local Moran index of high-quality tourist attractions in Shandong Province.

Taking its 16 administrative districts as the basic spatial unit, ArcGIS10.8 is used to calculate the global Moran’s I of the spatial distribution of high-quality tourist attractions in Shandong Province, whose value is 0.87 and normal statistic Z value is 21.83, greater than the critical value of 2.58 at the 0.01 confidence level. Furthermore, the test effect is more significant, indicating that the spatial distribution of high-quality tourist attractions in Shandong Province has a significant spatial autocorrelation and thus the overall distribution of its clustering characteristics. Calculating the local Moran’s I of high-quality tourist attractions (Fig 2) indicates that Qingdao is the only high-high agglomeration area, showing that Qingdao and its neighboring cities, such as Weifang and Yantai, have more high-quality tourist attractions and thereby form an advantageous area for tourism industry clustering. Heze is the only low-high agglomeration area with a large gap in the development level of its tourism industry.

**Fig 3:**
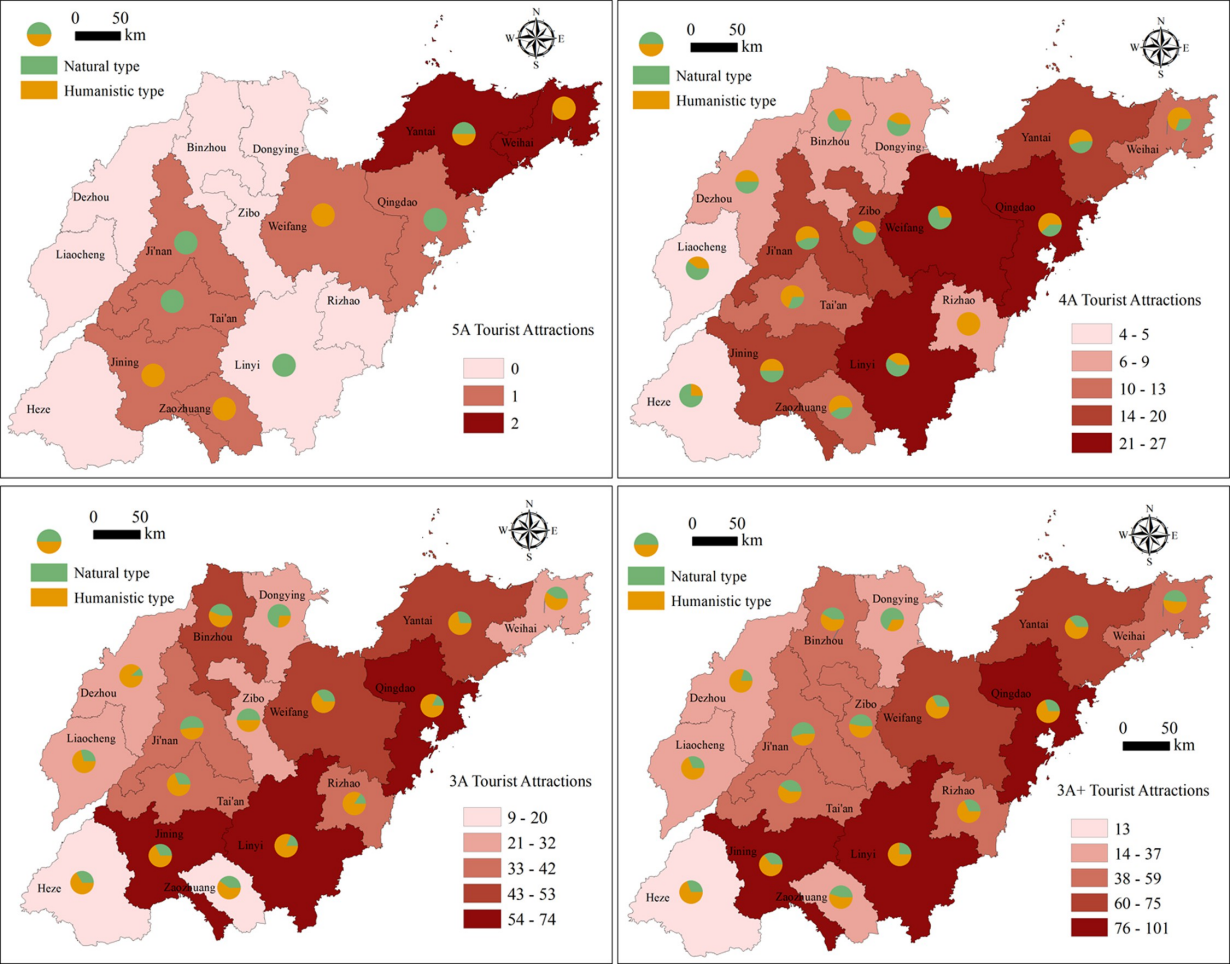
The numbers and types of tourist attractions in 16 cities in Shandong Province.

This study is based on the *Tourism Resource Classification*, *Survey and Evaluation (GB/T189722003)* promulgated by the state, the *Tourism Resource Classification System and Type Evaluation of China*, and the *Tourism Resource Classification and Evaluation Method*. Hence, it classifies the high-quality tourist attractions in Shandong Province into two categories, natural tourist attractions or humanistic tourist attractions, according to their scenic spot attributes and resource background, and it also analyzes the spatial distribution characteristics of these different types of tourist attractions. Based on Figs 2 and 3, the overall situation of high-quality tourist attractions in Shandong Province is clear; there are more in the east and central regions and fewer in the west. The spatial differentiation at the 5A level is not significant, and the numbers of natural and cultural tourist attractions are similar. The 4A level tourist attractions are concentrated in Qingdao, Weifang, Linyi and other places. Qingdao is a high concentration area with a relatively many natural tourist attractions. The 3A level tourist attractions have an obvious spatial differentiation, forming the Jining high–low cluster area and Zaozhuang and Heze low–high cluster areas. These tourist attractions are also primarily humanistic. Specifically, with the reduction in the concentration of tourist attractions, the number of humanistic tourist attractions has increased significantly, accounting for 62.56% of the total. Among the different cities, Qingdao has the most high-quality tourist attractions, and Heze the fewest. Indeed, the number of high-quality tourist attractions in Qingdao and its surrounding cities is over 50, and the number of high-quality tourist attractions in Jining City, which is connected to Heze, is 7 times higher, forming the obvious Qingdao high–high cluster and Heze low–high cluster.

#### 3.1.3 Kernel density estimation

**Fig 4:**
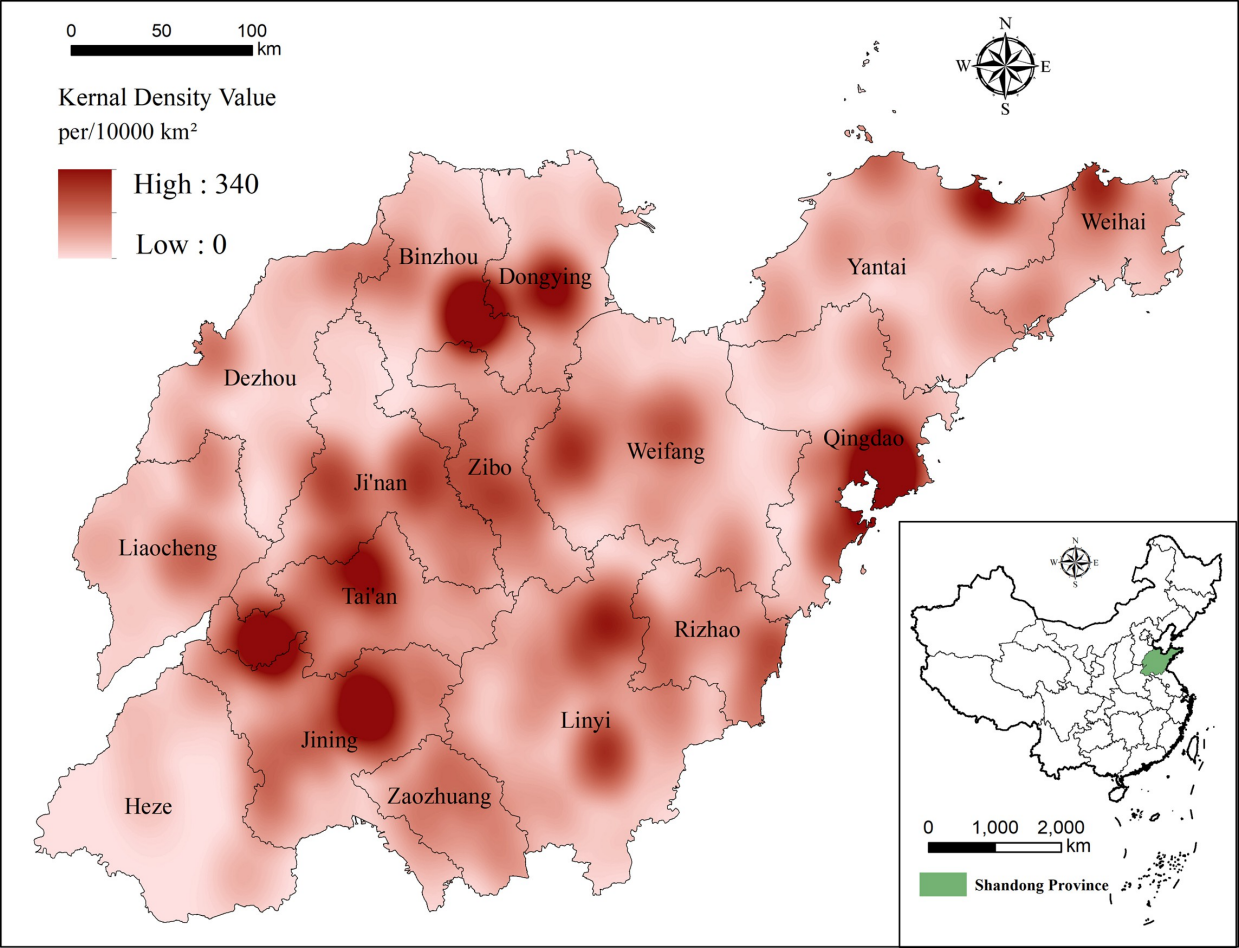
KDE of high-quality tourist attractions in Shandong.

ArcGIS10.8 is used to analyze the core density of 892 high-quality tourist attractions in Shandong Province. According to the relationship between area range and number of tourist attractions in Shandong Province, four search radii, 200 meters, 400 meters, 600 meters and 800 meters, are selected as the scale of bandwidth to determine their density field. There are too many first-level high-density areas below 400 meters and too many and few high-density areas above 600 meters, but the nuclear density cold and hot spots between 400 and 600 meters are relatively stable. This is representative, whereby the search radius is selected as 500 meters. These results are shown in Fig 4.

**Fig 5:**
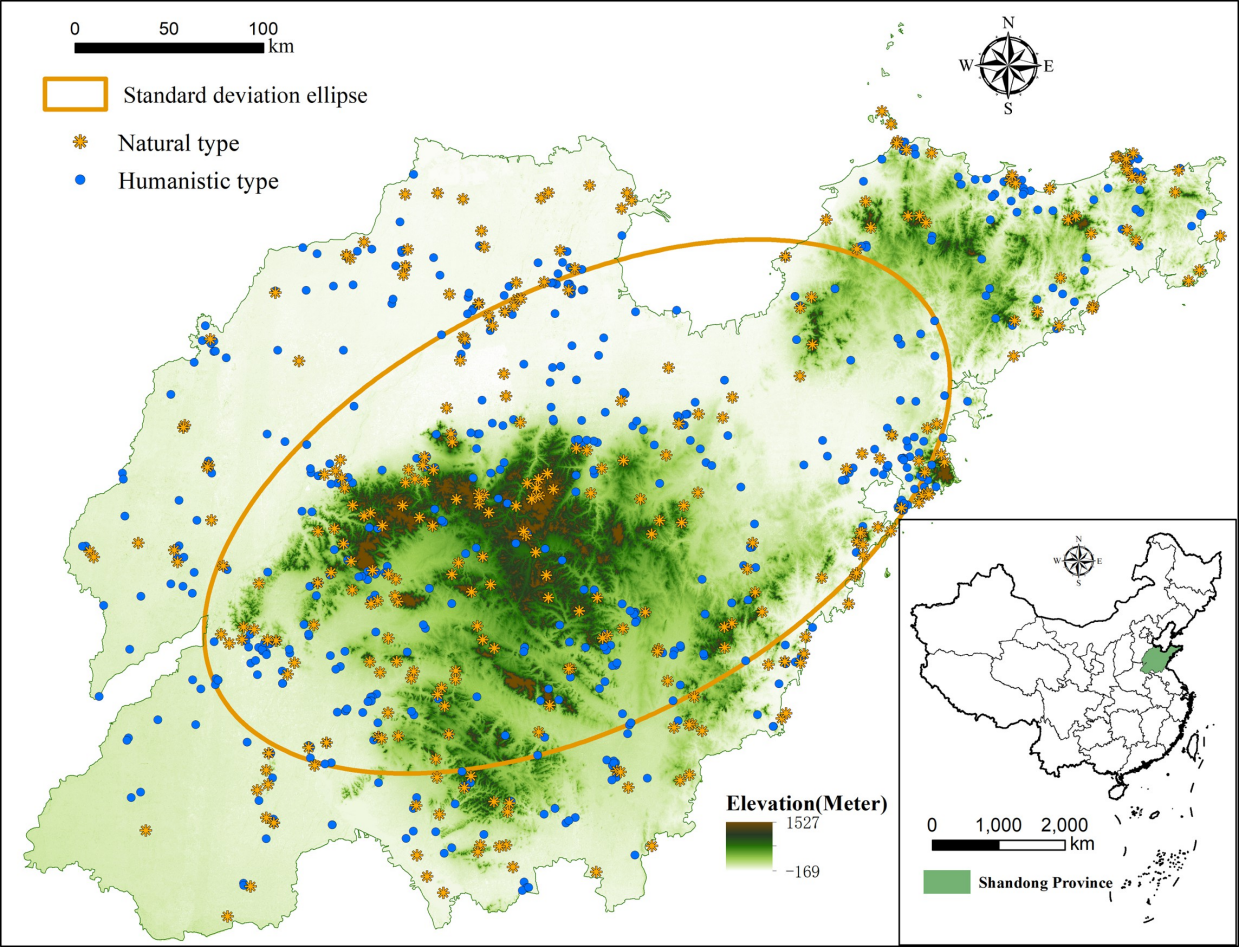
Coupling the standard deviation ellipse and topography in high-quality scenic areas.

Fig 4 shows that the distribution density of high-quality tourist attractions in Shandong Province generally shows the characteristics of "more in the east and central part and fewer in the west". Among them, the Jining–Jinan–Taian–Zibo–Binzhou–Dongying–Weifang–Linyi–Rizhao–Qingdao high-density areas interconnect to form a tilted "N" shape. Further analysis shows that the highest density of distribution of high-quality tourist attractions is in Jining, Qingdao, Tai’an, Dongying and Binzhou, with a distribution density of 185–340 per 10,000 km^2^. Zibo, Weifang, Zaozhuang, Linyi, Weihai and Yantai have the next highest distribution density, with values reaching 108–184 per million km^2^. Heze, Liaocheng and Dezhou have smaller distribution densities, all below 64 per million km^2^. Combining these results with those in Fig 5 demonstrates that the spatial distribution of high-quality tourist attractions is more depend on topography and that the three areas with higher densities are the three highest mountains in Shandong Province: Mount Tai, Mount Meng, and Mount Laoshan. The highest-density agglomeration center is at the junction of Jining-Tai’an, with the southern part of the subdensity center extending to Jinan and Zaozhuang and the northeastern part to Zibo and Weifang, where it connects with the Binzhou-Dongying agglomeration center. The coastal agglomeration center forms a relatively closed high-density distribution area around Jiaozhou Bay. Although the northern part is not directly connected to Weihai and Yantai, the subdensity center is distributed along the coast, and the seaside tourism belt, extending from Weihai and Yantai in the north and connecting Qingdao to Rizhao, is gradually taking shape.

#### 3.1.4 Standard deviation ellipse analysis

The standard deviation ellipse of the high-quality tourist attractions in Shandong Province is calculated, and the resulting spatial distribution is shown in Fig 5. As many as 613 high-quality tourist attractions are in the standard deviation ellipse, accounting for 68.7% of the total number, and the core density centers are also in the standard deviation ellipse, showing strong spatial agglomeration. Further analysis reveals that 177 natural and 347 cultural tourist attractions are located inside the standard deviation ellipse, accounting for 55.31% and 60.66%, respectively. The length of the long axis is 113.8 km, the length of the short axis is 81.9 km, and the ratio is 1.39:1, indicating that the spatial distribution of high-quality tourist attractions in Shandong Province is obvious, with the most distributed in the southwest‒northeast direction and the least in the northwest‒southeast direction. The latitude and longitude of the center of the standard deviation ellipse (118.42E, 36.35 N) and the geometric center of Shandong Province (118.16E, 36.35 N) are close to coinciding, indicating that the centripetality of high-quality tourist attractions in Shandong Province is obvious.

### 3.2 Factors influencing high-quality tourist attraction distribution

#### 3.2.1 Tourism resource endowment

Tourism resources are the precondition for developing tourist attractions, and the quality of the former has a strong positive correlation with the latter’s ratings. The high-quality tourism attraction resources in Shandong Province are mainly concentrated in the low mountain areas and foothills, on the coastal areas, and in cities renowned for their historical and cultural heritage. This study thus next examines how their geographic features and landscapes, historical and cultural heritage, and climatic conditions influence the spatial distribution of tourist attractions.

*(1) Geographic features and landscapes*. Geographic features and landscapes provide intrinsic support for tourist attractions. Various landscapes enhance the allure of attractions and increase their aesthetic value. A combination of diversified landscapes, roads, and architecture creates attractions with distinguishing features. Given the limitations that geographic elements and landscapes impose on tourism resources, the topographic map of Shandong Province is overlaid on the map of its high-quality tourist attraction distribution (Fig 5).

Shandong has diverse geographic features and landscapes, consisting mainly of low mountains, foothills, and plains. The variations in landscapes across regions is significant. Central Shandong, South Shandong, and the Jiaodong Peninsula mainly feature low mountains and foothills. These regions account for 61.3% of Shandong’s total land area; however, as of the end of 2020, they boasted 90.9% of its 5A tourist attractions, 78.1% of its 4A tourist attractions, and 67.9% of its 3A tourist attractions. The N-shaped high-density distribution that was revealed in the kernel density estimation also roughly aligns with the distribution of low mountains and foothills in the central and southern regions. Moreover, the high-density centers are in the mountainous areas of the Central Region, South Region, and Jiaodong Peninsula. The landscapes of Northwest and Southwest Shandong are dominated by plains and lack variety. Comprising 38.7% of the province’s land area, these two regions only account for 25.1% of its high-quality tourist attractions. In summary, geographic features and landscapes constitute a limit on tourism resources and the distribution of high-quality tourist attractions and therefore have far-reaching impacts on tourism resource exploitation and tourist attraction evolution.

*(2) History and culture*. To a large extent, the designation of high-quality tourist attractions relies on naturally bestowed unique scenes or deep-rooted historical and cultural heritage. Cultural tourism resources can also be called humanistic tourism resources, referring to the natural features, historical and cultural heritage, or social phenomena that objectively exist in a certain geographical space, have high cultural value, and can attract tourists. Shandong has a strong cultural tourism sector and has historically been considered “a state of ceremonies and a thousand miles of fertile soil”. The current tourism branding of Shandong, “Friendly Shandong”, has gained strong momentum nationwide. Confucianism, which originated in Shandong, still has a profound influence on its peoples’ work, life, and ethical pursuits.

The historical and cultural tourism resources of Shandong are highly concentrated in 10 National Famous Historical and Cultural Cities, including Qufu, Qingzhou, Jinan, and Tai’an. These 10 cities have 7 5A tourist attractions, accounting for 63.6% of Shandong’s 5A tourist attractions; 91 4A tourist attractions, accounting for 40.6% of its 4A attractions; and 254 3A tourist attractions, accounting for 38.7.3% of its 3A attractions (Table 4). Of the eight cultural tourism zones in Shandong, six are associated with these 10 cities: the Oriental Study Cultural Tourism Zone, jointly developed by Jinan, Tai’an, Qufu, and Zoucheng to integrate their geographic advantages and cultural resources; the Qi Culture Tourism Zone, centered in Linzi; the Marine Culture Tourism Zone, developed mainly around the coastal cities Qingdao, Penglai, and Yantai; the Folk Culture Tourism Zone, with Weifang and Qingzhou at the center; the Water Margin Culture Tourism Zone, represented by Liaocheng and Heze; and the Canal Culture Tourism Zone, mainly represented by Liaocheng, Zaozhuang, Jining, and Zoucheng. Furthermore, of the province’s 10 cultural tourism destination brands, seven are connected to these 10 cities. Among these attractions, those that are well developed and well known are the most substantial and form cultural attractions, such as historical and cultural heritage entities, museums, and memorial places honoring the Chinese revolution. Therefore, history and culture have influenced the development of these cultural tourism zones and destinations and have played a critical role in the distribution of cultural tourism resources and high-quality tourist attractions.

**Table 4:** Famous historical and cultural cities in Shandong and related high-quality tourist attractions.

Famous Historical and	Number of 5A	Number of 4A	Number of 3A	Total
Cultural Cities	Tourist Attractions	Tourist Attractions	Tourist Attractions	
Qingdao	1	26	74	101
Jinan	1	16	42	59
Tai’an	1	13	42	56
Yantai	1	20	27	48
Liaocheng	0	5	27	32
Zoucheng	0	3	15	18
Qufu	1	2	10	13
Qingzhou	1	3	9	13
Penglai	1	2	5	8
Linzi	0	1	3	4

Further analysis indicates that the combination of landscapes and historical and cultural heritage has been a strong driving force in the development of Shandong’s tourist attractions. Of the 10 National Famous Historical and Cultural Cities, eight are in the mountainous areas and foothills in the Central and South Regions and the Jiaodong Peninsula. Thus, resource endowment is a limiting factor in the distribution and evolution of high-quality tourist attractions.

**Fig 6:**
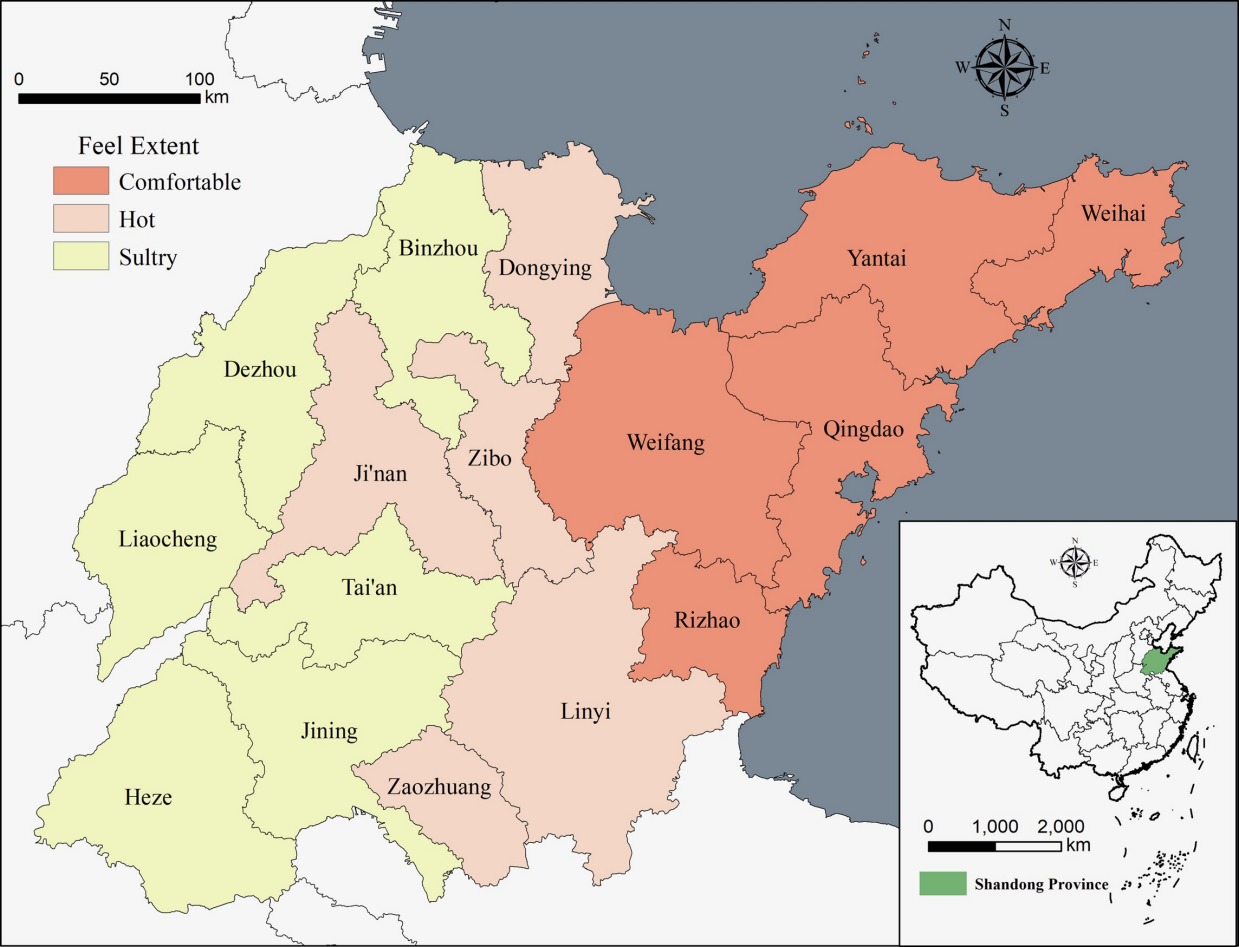
Shandong Province’s summer climate comfort rating in 2020.

*(3) Influence of climatic conditions*. Meteorological and climatic factors are not only an integral part of the tourist landscape but are also important environmental factors that affect the flow of visitors. Shandong Province experiences an uncomfortable period for tourism in the winter half of the year, when the wind efficiency index in all cities is between -800 and -600; people feel cold and uncomfortable in this environment, which is not suitable for tourism activities. The most comfortable period for travel is May to mid-September, which mainly falls in the summer half of the year. In Yantai, Qingdao, Weihai, Rizhao and Weifang, the environment is Comfortable. Jinan, Zibo, Linyi and other south-central regions, the environment is hot, while in Heze, Dezhou, Binzhou and other western cities, the environment is sultry, both of which are uncomfortable (Fig 6). The Temperature and humidity index and Wind efficiency index effectively reflect the comprehensive degree of human perception of the environment and provide strong support for the interpretation of climate comfort in different regions of Shandong Province. The seaside area has the advantage of better climate resources; during the summer, its climate is more comfortable than those in the south and west of Central Shandong. Although short, the peak season for tourism in the seaside area is when its climate is the most comfortable, when the summer wind regulates the temperature, which makes the seaside an excellent summer destination. Jiaodong Peninsula has both low hills and marine scenery compared to the mountainous hilly areas of south-central Shandong, and the seaside areas of Qingdao, Yantai and Weihai are more attractive to tourists. Meanwhile, due to its proximity to Japan and Korea, its domestic and foreign source markets are stable and sufficient, whereby the demand for its number of high-quality tourist attractions is greater. Climate comfort affects the development of the tourism industry, and desirable climate resources positively promote tourist attractions. Compared to the inland areas, then, the seaside area has created more high-quality tourist attractions to meet the needs of its tourism market.

#### 3.2.2 Socioeconomic development

The state of socioeconomic development determines the development standards and trends of tourist attractions and is a major force in the distribution thereof. In economically developed areas, industries closely related to tourism, such as transportation, accommodations, health care, restaurants, and education, are usually well established and can drive the sustainable growth of the tourism industry. We therefore use the 2020 GDP of the 16 focal cities as indicators of economic development and for correlation analysis in SPSS. These results indicate that the Pearson correlation coefficient between tourist attractions and a city’s GDP is R = 0.64**, greater than 0.6 and smaller than 0.8 (P value 0.001), demonstrating that these two variables have a strong positive correlation and that a city’s GDP has an important impact on the distribution of tourist attractions. As shown in Table 5, Qingdao and Yantai are thus high in both tourist attractions and GDP, while Heze and Liaocheng are low in both. Ten cities, including Qingdao, Yantai, and Weifang, have a relatively strong correlation between GDP and the number of tourist attractions. In total, the cities with a strong correlation between the two indicators account for 81.3% of all cities, indicating that the state of economic development has a significant impact on the development of tourist attractions.

**Table 5:** Number of high-quality tourist attractions and GDP of prefecture-level cities in Shandong.

City	Number of Above 3A level Tourist Attractions	City GDP (RMB 0.1 Billion)
Qingdao	101	12400.56
Linyi	98	4805.25
Jining	88	4494.31
Yantai	75	7816.42
Weifang	71	5872.17
Jinan	59	10140.91
Binzhou	57	2508.11
Tai’an	56	2766.46
Zibo	47	3673.54
Rizhao	47	2006.43
Weihai	44	3017.79
Dongying	37	2981.19
Dezhou	34	3078.99
Zaozhuang	33	1733.25
Liaocheng	32	2316.84
Heze	13	3483.11

#### 3.2.3 Transportation

**Fig 7:**
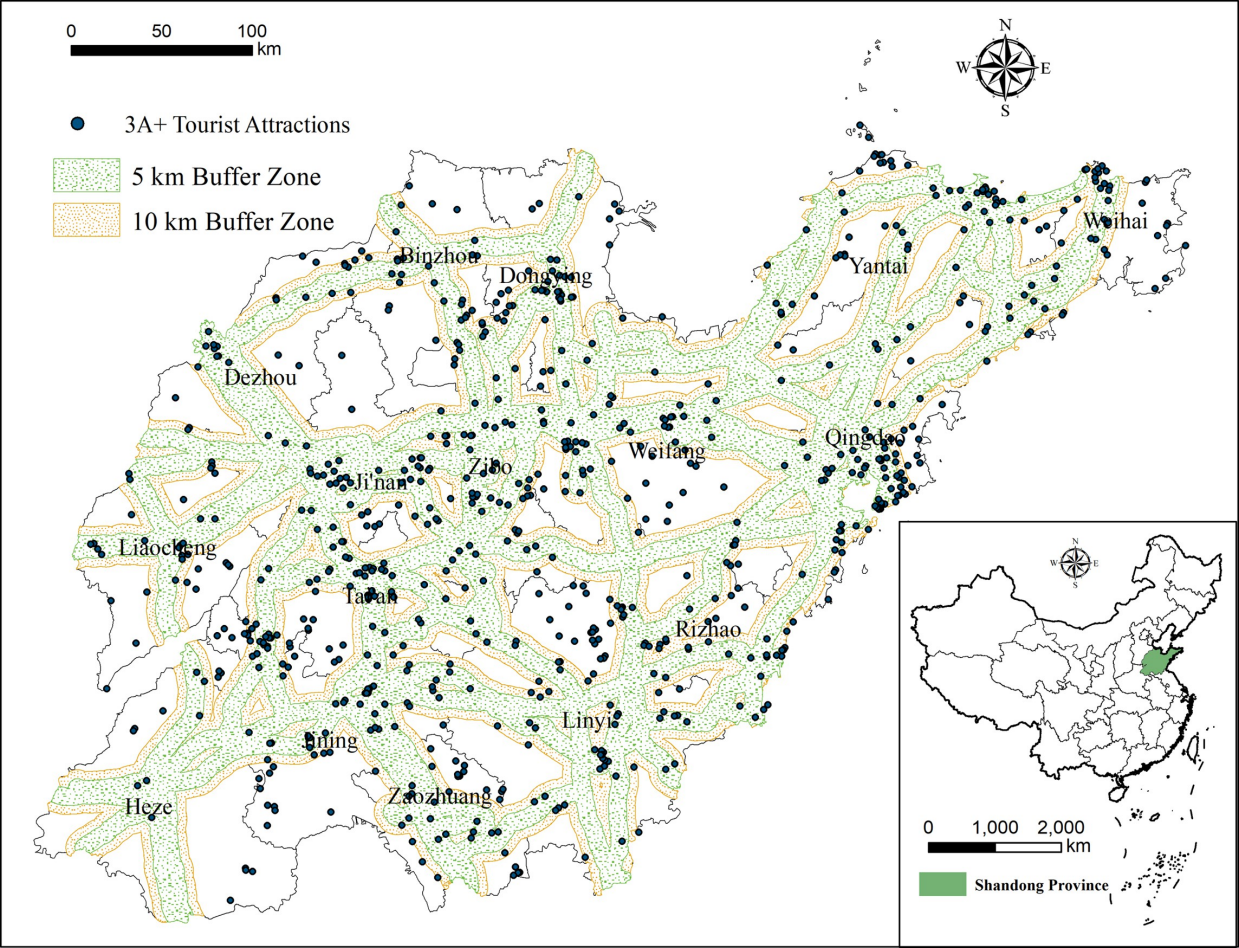
Major transportation routes and 4A+ tourist attractions in Shandong.

Transportation is an important measurement of the quality of tourist attractions, as it affects the psychological distance between attractions that tourists perceive and thus influences their travel decisions [42]. As of the end of 2020, Shandong had a highway network of 286,814 km including 7,473 km of expressway. The total length of the railway in Shandong was 6,881 km, including 2,110 km of high-speed rail. Shandong therefore has a convenient transportation network that profoundly affects people’s travel plans. This network ensures complete connectivity between tourists and attractions. Hence, we use the buffer analysis tool in ArcGIS10.8 to construct 5-km and 10-km buffer zones along the national highways, expressways, and railways within Shandong Province, thereby forming two buffer zones with widths of 10 km and 20 km, respectively. Regarding the number of tourist attractions in each buffer zone (Fig 7), within the 10-km buffer zone, there are 469 high-quality tourist attractions, 52.58% of all attractions; in the 20-km buffer zone, there are 635 high-quality tourist attractions, 71.19% of total attractions. These results indicate that transportation has a major impact on the spatial distribution of tourist attractions.

### 3.3 Optimization measures for the spatial layout of high-quality tourist attractions in Shandong Province

Shandong has many high-quality tourist attractions, and they are spatially clustered. The number of attractions varies significantly from region to region. Accordingly, to enhance the sustainable development of Shandong’s tourism industry, the province may be able to optimize this distribution in the following three ways:

(1) Strengthen regional tourism cooperation and broaden the Qingdao-Jinan traffic corridor. The differences and complementarities of tourism resource types and cultural heritage are the basis for cooperation across different regions, whereby Jinan and Qingdao have become the twin core cities of tourism in Shandong Province due to their long-term development based on a significant regional radiation drive. The coastal city tourism area with Qingdao as the core has successfully created a fairyland coastal cultural tourism destination with a stable domestic and foreign source market. Jinan, as the core of the cultural tourism circle around the provincial capital including Zibo, Weifang, Tai’an, Jining and other cities has many cultural heritage sites and mountain, river and lake tourist attractions and has created a "sacred land of the East, the peace of Mount Tai, the former capital of Qi, the spring city of Jinan" and other cultural tourism destinations. Standard deviation ellipse analysis of Jinan-Qingdao indicates the direction of high-quality tourist attraction clustering in Shandong Province, which reflects the creation of an exclusive tourism channel between Qingdao and Jinan, improving transportation facilities, increasing regional accessibility, exploiting the many tourism centers in Jiaodong Peninsula, increasing passenger flow to the central and southern regions of Shandong, promoting the union of different types of tourist attractions, and creating more tourism revenue.

(2) Optimize the spatial layout of tourism products and construct new tourism functional areas. Efforts should be made to accelerate the construction of the "N" type high-density tourism product distribution belt based on the integration of resources, to give full play to the advantages of different types of tourism resources with regard to the high-density belt, and to more deeply explore the cultural connotations and organic combinations of different areas of tourism product clusters. The high-density area covers tourism brands such as Mountain Tai, Confucius’ Hometown, Confucian Style Canal, Yellow River into the Sea, Qi State Capital, Chinese Dragon City, Affectionate Yimeng and Seaside Scenery. This area should thus not only play a leading role among the regionally advantageous tourism clusters and build new tourism destinations but also divide tourism function partitions according to the characteristic resource advantages of the different municipalities, promote resource integration, improve brand awareness, differentiate the positions of regional development strategies, and realize cross-regional integrated development. Regions with a weak economic development base should implement the tourism development strategy of " The eastern region of Shandong Province drives the development of the western region ", set reasonable development goals, cultivate model tourist attractions, give full play to the advantages of their borders with other provinces, and formulate preferential policies to attract investment and cooperation from neighboring provinces to achieve rapid tourism development.

(3) Coordinate the development of tourism and urbanization. Tourism is an important driving force in the level of urbanization. Urbanization is an important support and guarantee for the development of tourism. High-quality tourist attractions in Shandong Province are mostly dependent on prefecture-level city, whereby the city’s brand, culture and service quality greatly affect each visitor’s perception and experience. Decision-makers should therefore organically unite town development planning and tourism industry planning, combine the resource advantages of high-quality tourist attractions, improve urban functional zoning around core scenic areas, realize reasonable layouts and orderly construction, ensure high-quality development, strengthen the growth pole role of large cities, incorporate small town tourist areas into the development circle, foster coordinated development across urban areas, small towns, urban‒rural areas and villages, and enhance overall attractiveness. The tourism industry has both industrial correlations and strong driving forces; it can thus obtain development funds through investment and consumption efforts, strengthen the flows of people, logistics and capital, drive the development of related industries, improve tourism-specific facilities, expand tourism demand and achieve greater social and economic benefits.

## 4. Conclusions and discussion

### 4.1 Conclusions

This study employs ArcGIS10.8 software and the analytical techniques of the nearest neighbor index (NNI), the standard deviation ellipse, spatial autocorrelation analysis, and kernel density estimation (KDE) to study the spatial distribution of high-quality tourist attractions in Shandong Province. Accordingly, we can draw the following conclusions:

1. High-quality tourist attractions in Shandong Province show a typical spatial distribution of the clustering state. The direction of clustering is southwest‒northeast, and the distribution range of the standard deviation ellipse mainly covers the south of Central Shandong and Jiaodong Peninsula, the main distribution area of high-quality tourist attractions.
2. Analysis of the spatial correlation of high-quality tourist attractions in 16 cities in Shandong Province reveals that Qingdao is the only high-high concentration area and that Qingdao and its neighboring cities collectively form a superior area for seaside tourism in Shandong Province. Heze is the only low-high agglomeration area with the least number of high-quality tourist attractions. Moreover, high-quality tourist attractions in Shandong Province show a typical and uneven spatial distribution.
3. Nuclear density analysis shows that the high-quality tourist attractions are spatially connected to the "N" type high-density belt of Jining-Binzhou-Linyi-Qingdao. Three agglomeration centers exist at the Jining-Tai’an intersection, the Binzhou-Dongying intersection, and on the coast of Qingdao around Jiaozhou Bay; the subdensity area is basically coincident with the distribution of the low mountainous hilly areas in Central Shandong, Southern Shandong and Jiaodong Peninsula.
4. The spatial distribution of high-quality tourist attractions is more dependent on resource endowment, topography and landscape. Historical, cultural and climatic resources are the intrinsic factors influencing scenic spot development; that is, the more undulating the terrain, the richer the cultural heritage, and the higher the grade, the greater the number of tourist attractions in areas with higher climatic comfort is. The developed regional economy and convenient transportation conditions are thus external guarantees for the development of tourist attractions, which can produce stable source markets for tourist attractions and play a greater role in promoting the spatial layout thereof.

### 4.2 Discussion

This study has carried out a detailed analysis of the spatial distribution and factors influencing high-quality tourist attractions in Shandong Province—selected terrain, climate, history and culture, economy, transportation and other factors that affect the spatial distribution of high-quality tourist attractions—and proposed optimization measures for the optimization and upgrading of its regional tourism. However, the selection of influencing factors needs to be more comprehensive, such as by considering social, economic, and natural resource factors, e.g., government policy support, social enterprise investment, number of star-rated hotels, and river water systems. In contrast to other scholars’ analyses of the spatial distribution characteristics and factors influencing tourist attractions in different provinces in China, this study uses mathematical models to evaluate the climate comfort of cities in Shandong Province and analyzes the impact thereof on tourists’ choice of destination, which is innovative. Moreover, it shows that this environment not only provides tourism resources and important support in the construction of tourist attractions but also has a greater impact on the sustainable development thereof. Exposure to environmental pollution has a definite, negative impact on the sustainable development of the tourism industry and has received increasing attention from scholars [43]. Shandong Province has both natural scenery and cultural heritage, and tourism has become an essential means for its regional economic growth. Therefore, focusing on the coordinated promotion of tourist attraction development and ecological environmental protection has become the focus of research on the future development of tourist attractions.

While this study adopts the dichotomous method to classify tourist attractions, natural and human resources can be further subdivided according to their attributes. For some tourist attractions, there is a certain subjectivity when classifying them according to their resource background; for example, Mount Tai is a World Cultural and Natural Heritage site, and its classification into the natural category is based on tourist review websites such as Ctrip (www.ctrip.com), which provide tourist review information. Therefore, constructing a more complete index and more deeply analyzing the influence mechanism of the distribution of different types of tourist attractions will provide more possibilities for the development of cultural and tourism integration and tourism industry optimization.

## Supporting information

S1 Appendix List of 892 high-quality tourist attractions in Shandong Province. (XLSX)
